# Subset specification of central serotonergic neurons

**DOI:** 10.3389/fncel.2013.00200

**Published:** 2013-10-31

**Authors:** Marten P. Smidt, Johannes A. van Hooft

**Affiliations:** Center for Neuroscience, Swammerdam Institute for Life Sciences, University of AmsterdamAmsterdam, Netherlands

**Keywords:** serotonin, dopamine, differentiation, prosomere, development

## Abstract

The last decade the serotonin (5-hydroxytryptamine; 5-HT) system has received enormous attention due to its role in regulation of behavior, exemplified by the discovery that increased 5-HT tone in the central nervous system is able to alleviate affective disorders. Here, we review the developmental processes, with a special emphasis on subset specification, leading to the formation of the 5-HT system in the brain. Molecular classification of 5-HT neuronal groups leads to the definition of two independent rostral groups positioned in rhombomere 1 and 2/3 and a caudal group in rhombomere 5-8. In addition, more disperse refinement of these subsets is present as shown by the selective expression of the 5-HT1A autoreceptor, indicating functional diversity between 5-HT subsets. The functional significance of the molecular coding differences is not well known and the molecular basis of described specific connectivity patterns remain to be elucidated. Recent developments in genetic lineage tracing models will provide these data and form a major step-up toward the full understanding of the importance of developmental programming and function of 5-HT neuronal subsets.

## INTRODUCTION

Serotonin is one of the monoamine neurotransmitters in the brain and has a widespread innervation pattern. The importance of the serotonergic transmitter system is exemplified by the association of serotonergic activity with psychiatric disorders like depression, and specific drugs aimed at changing the serotonergic tone, as selective serotonin reuptake inhibitors (SSRIs), are widely used in the clinic to alleviate such psychiatric disorders. Serotonergic neurons are generated in the central nervous system (CNS), born between E10.5 and E12.5 in the mouse (Pattyn et al., 2004), and make up the anatomical locations designated as B1–B9 in the adult brain (reviewed in Goridis and Rohrer, 2002 and refined in Alonso et al., 2013). These neurons are identified by the enzymes that produce serotonin through the hydroxylation (Tryptophan hydroxylase, Tph2) of tryptophan to 5-hydroxytryptophan and the subsequent decarboxylation (L-Aromatic amino acid decarboxylase, Aadc) to produce serotonin. The latter enzyme is shared with dopaminergic, noradrenergic and adrenergic neurons, since it also catalyzes the decarboxylation of L-dopa to dopamine.

It has become apparent that prenatal and early postnatal exposure to SSRIs can have an important influence on the development of the CNS, which can result in lifelong modifications of behavior (Ansorge et al., 2004,^2008^; Noorlander et al., 2008; Smit-Rigter et al., 2012). This influence seems a logical consequence of the fact that the pharmacological target, the serotonin reuptake transporter, Sert, is expressed during development (reviewed in Daws and Gould, 2011) and may therefore influence the 5-hydroxytryptamine (5-HT) tone during developmental processes. These data highlight the notion that 5-HT is not merely a classical neurotransmitter, but has neuromodulatory and trophic actions which are essential for proper brain development (reviewed in Daubert and Condron, 2010; Homberg et al., 2010). The essential role of 5-HT during development and in the adult has therefore raised a long-standing interest in the developmental programs that define 5-HT neurons (reviewed in Kiyasova and Gaspar, 2011; Deneris and Wyler, 2012). Interestingly, the data from genetic studies in mice have shown that the molecular programming over the rostral caudal axis, of the 5-HT neuronal containing regions in the hindbrain, is not equal. In this review we focus on these molecular distinctions and try to propose subset specific programs in the development of 5-HT neurons.

## DEVELOPMENTAL PROCESSES THAT DETERMINE THE PERMISSIVE REGION FOR SEROTONERGIC NEURONAL DEVELOPMENT

An essential first step in providing cellular diversity is the subdivision of the developing CNS in longitudinal and transverse domains which are specified through specific gene expression patterns. The longitudinal domains are designated: floor plate, basal plate, alar plate, and roof plate. The transverse domains along the anterior/posterior (A/P) axis lead to the following domains in a rostral to caudal order: telencephalon, rostral diencephalon, prosomer 3-1, midbrain, and hindbrain (Puelles and Rubenstein, 2003). Since serotonergic neurons are born in the hindbrain rhombomere 1–8 region (reviewed in Deneris and Wyler, 2012) the anterior border of the permissive region is formed by the mid/hindbrain border; the isthmic organizer. The hindbrain A/P segmental origin is coded by a combinatorial code of Hox gene expression (reviewed in Alexander et al., 2009). The most rostral expression boundary is formed by the expression of Hoxa2 at the R1/R2 boundary, leaving the rhombomere 1 segment without influence of Hox gene expression. The caudal position of serotonergic permissiveness is coded by the presence of high local retinoic acid (RA) synthesis inducing Hoxa/b/d4 toward R7/8.

The origin of the 5-HT cell groups rely on floor plate signals as Shh. The different response of cells to go into serotonergic instead of other monoaminergic cell types differentiation relies on specific Fgf signals. The combination of early signaling of Shh and Fgf4 leads to the correct patterning of the region that later produces neurons with the rostral serotonergic identity (Ye et al., 1998). Moreover, Fgf4 and in addition Fgf2 were able to ectopically induce the 5-HT phenotype. Through elegant *ex vivo* experiments in rat and chicken, Farkas et al. (2003) have shown that Tgf-β is an additional signaling component, essential for the early Shh signaling and subsequent induction of floor plate derived neuronal systems. This signaling positions Tgf-β together with Fgfs and Shh (Hynes et al., 1997; Ye et al., 1998) central in defining the local molecular signaling in order to enable the progress of serotonergic differentiation within the permissive region.

Early molecular coding of the ventricular zone influences the fate of newborn neurons at specific dorsal/ventral positions along the A/P axis (Craven et al., 2004). The coding is generated by early players that are involved in early instructive signals during CNS patterning. Therefore, specific transcription factors expressed in the ventricular zone instruct newborn neurons for their early differentiation steps into the serotonergic phenotype. In the following sections we will discuss the general and subset specific transcriptional programs that define and generate specific serotonergic neuronal clusters.

## SEROTONERGIC NEURONAL SPECIFICATION

### GENERAL MOLECULAR PROGRAMMING OF SEROTONERGIC NEURONS

**FIGURE 1:**
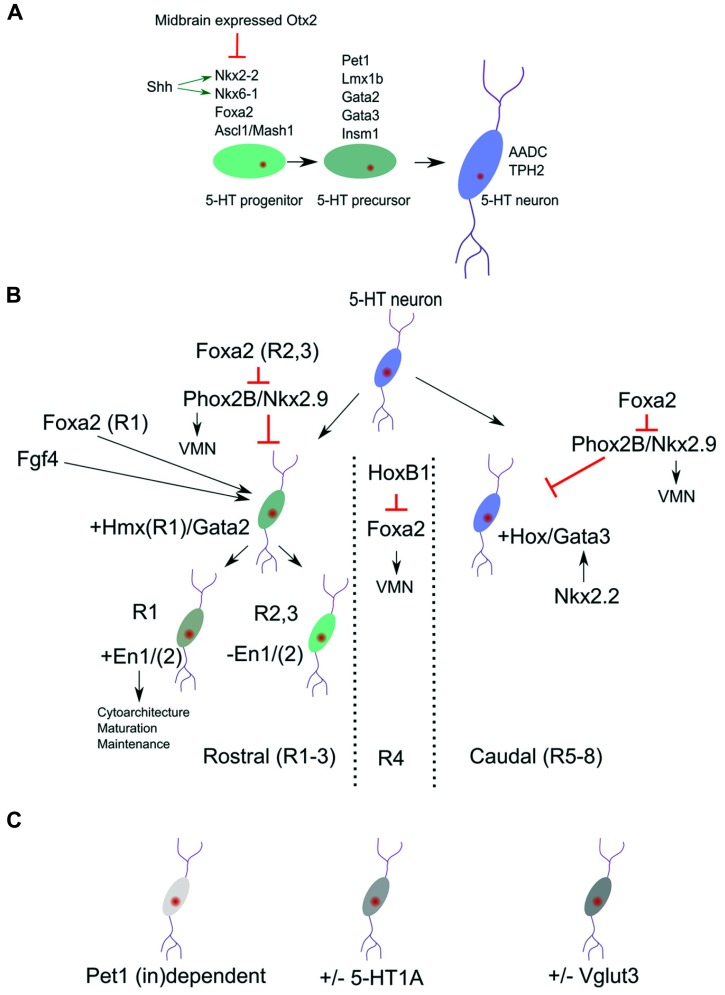
**Molecular identity of 5-HT neuronal subgroups.** **(A)** Definition of broads programs of 5-HT neuronal specification. **(B)** The general pool of 5-HT neurons is molecularly divided in a rostral and caudal subgroup. These subgroups are identified by the presence of *Hmx/Gata2* and *Hox/Gata3* genes. The most rostral group can be subdivided by the presence or absence of the homeodomain genes Engrailed 1 and 2 (En1/2). **(C)** Next to the position related programming differences **(B)** more disperse transcription factors dependence (Pet1) and 5-HT subset markers exist in the 5-HT neuronal cluster.

The genetic network of gene activation leading to the appearance of 5-HT neuronal groups has been studied in detail and have led to the definition of a specific transcription factor program (recently reviewed in Kiyasova and Gaspar, 2011; Deneris and Wyler, 2012). Here we will recapitulate the most important events that lead to the generation of 5-HT neurons (**Figure 1A**; for a complete model see Deneris and Wyler, 2012) as parts of this programming is also used to generate 5-HT neuronal diversity among the different 5-HT subsets (see below). As mentioned above, in a permissive region in the rostral hindbain region signaling events lead to the induction of critical early activators as Foxa2, Ascl1, Nkx2.2, and Nkx6.1.

The early expression of Foxa2 is required to suppress the expression of Phox2b and thereby initiate a switch from visceral motor neuron (VMN) programming toward 5-HT programming. Importantly, conditional deletion of Foxa2 *in ovo* in the posterior hindbrain did show an equal distributed diminishing of all 5-HT neurons without affecting the expression of Nkx2.2 (Jacob et al., 2007), suggesting that Foxa2 acts as a separate essential activator for the serotonergic cell-fate in all 5-HT neuronal clusters.

As a result of the presence of high concentration of the signaling molecule Shh (reviewed in Tannahill et al., 2005), a medial ventral domain in the rostral hindbrain starts to express the critical activator of the 5-HT lineage, Nkx2.2. Analysis of Nkx2.2 ablated mutants showed that serotonergic neurons at rhombomere2 level are ablated (R1 level is spared) as a consequence of a ventral to dorsal shift of programming at this A/P position (Briscoe et al., 1999). In addition, the essential role for Nkx2.2 in specifying the 5-HT phenotype was underscored by the fact that ectopic 5-HT neurons could be detected in the midbrain in Otx2 mutants where, as a consequence of changed Otx2 dose, Nkx2.2 is ectopically upregulated (Vernay et al., 2005). These data suggest that other essential activators for the 5-HT lineage are present in the midbrain and that Nkx2.2 upregulation is enough to initiate 5-HT specification programs.

It was shown that Nkx2.2 has to work together with other factors as Ascl/Mash1 in programming neurons toward the 5-HT phenotype. Analysis of Ascl1/Mash1 mutants has indicated that Ascl1 together with Nkx2-2 is involved in activating the 5-HT specification factors Lmx1b, Pet1, and Gata3 (Pattyn et al., 2004). Moreover, it has been suggested that Ascl1 binds the promoter of Insm1 and thereby activates this factor which was shown to be essential for the activation of the 5-HT synthesis gene Tph2. In Isnm1 ablated mice, the 5-HT neurons are generated but show lower expression of the 5-HT differentiation factors Pet1, Lmx1b, and Gata2, in addition to the failed Tph2 presence (Jacob et al., 2009). These data underscore the importance of Insm1 and suggest that its activation is downstream of Ascl1, besides the cooperation with Ascl1, in activating the full 5-HT phenotype.

In parallel to the above described activation of Gata2/3 by the Nkx2.2/Ascl1/Insm1 program, is the activation of the Lim homeobox gene Lmx1b, which is co-expressed in all 5-HT neurons (Ding et al., 2003). It has been suggested that Lmx1b acts as an intermediate step in the terminal differentiation between Nkx2.2 activity and activation of Pet1. Interestingly, Lmx1b ablation studies have shown that all 5-HT neurons rely on this transcription factor for their normal developmental program (Cheng et al., 2003). In these Lmx1b mutants the expression of Pet1, driving the 5-HT terminal differentiation markers, is eventually lost, leading to the absence of 5-HT phenotypic characteristics. Moreover, in Pet1 mutants the expression of Lmx1b is unaffected, suggesting that Lmx1b is positioned upstream of Pet1 in the genetic cascade. However, close examination of the developmental program in Lmx1b mutants suggest that the initial activation of Pet1 (E11.5-E12.5) in these mutants is unaffected (Cheng et al., 2003). This indicates that Lmx1b is not required for initial Pet1 activation, but in maintaining the Pet1 expression. Conditionally ablated Lmx1b mice [driven by Cre under the control of Pet1; (Zhao et al., 2006)] displayed a normal initial generation of 5-HT neurons. At E12.5 the amount of 5-HT neurons was markedly decreased and by E14.5 almost all neurons are lost as measured by 5-HT markers as Tph2 and Sert. These data suggest that Lmx1b is essential for maintaining the expression of Pet1 and therefore the 5-HT phenotype. The essential step in terminal differentiation, inducing the 5-HT phenotype, is established, by the action of Pet1 (Hendricks and Francis, 1999; Hendricks et al., 2003; Liu et al., 2010). This Ets factor is expressed just before the cells are starting to display 5-HT production. In ablation studies it was shown that Pet1 is essential, since in its absence most 5-HT precursors fail to develop (see also below) and the cells lack the genes for synthesis, uptake, storage, and signaling of 5-HT. Besides these most general 5-HT neuronal specification programs, it has become apparent that molecular differences exist within the 5-HT neuronal population, indicating that subsets specific programming might exist which we will discuss in more detail below.

### PONTINE VERSUS MEDULLAR CODING OF 5-HT NEURONAL GROUPS

In most of the studies described above it was clear that not always all 5-HT neuronal groups were affected equally by ablation of essential transcription factors. This suggests that next to the overall existence of a general genetic program toward 5-HT neurons, other specific programming should be present that defines such distinctions.

Among the first emergence of subset specification is the division of rostral (pontine, R1 and R2/3) and caudal (medullar, R5-8) 5-HT clusters. The rostral cell group are located close to the caudal edge of the isthmus and the caudal cell group in the caudal myelencephalon which are divided by a region that does not contain any 5-HT neurons [branchial motor area; (Pattyn et al., 2004)]. This space devoid of 5-HT neurons located around rhombomere4 could be initiated through the activation of the 5-HT-program repressive transcription factor Phox2b, which is activated in this region through the local activation of Hoxb1 (Jacob et al., 2007 and reviewed in Alexander et al., 2009). This initial diversity is mimicked by the selective dependence of developing 5-HT neurons on the presence of the transcription factor Nkx2.2. In classic Nkx2.2 knock-out animals large groups of 5-HT neurons are ablated. However, a small dorsal group remains in these animals suggesting that they are not depending on Nkx2.2 activity for the presence and survival (Briscoe et al., 1999; Hendricks et al., 2003; Jensen et al., 2008). The activity of Nkx2.2 is combined with the presence of the forkhead box protein a2 (Foxa2) and repression of the VMN activators, paired like homeobox 2b (Phox2b) and Nkx2.9. This combinatorial activation leads to the repression of the VMN fate and appearance of 5-HT neurons in R2/3 and R5-8. The activation of 5-HT neurons in of rhombomere 1 seems to follow a direct activation pattern, instead of fate change from VMN to 5-HT, as exemplified by their 1~day earlier appearance during development (Jacob et al., 2007). These data suggest that Foxa2 has a dual function in 5-HT specification, one direct acting as an intrinsic determinant for 5-HT neuronal differentiation (all 5-HT groups) and second, indirect, through repression of the VMN fate in R2/3 and R5-8. In the region where no 5-HT neurons are generated, R4, the presence of HoxB1 represses the dorsal expansion of Foxa2 en thereby maintains the VMN fate in this region (Jacob et al., 2007; **Figure 1B**). It is currently unknown how exactly the selective independence of R1 5-HT neurons on Nkx2.2 function is programmed. It seems likely that this independence is linked to the difference between direct activation of the 5-HT program as present in R1 compared to the indirect activation via the VMN program as present in R2/3 and R5-8, and exemplified by the R1 absence of Hox activity (Wylie et al., 2010 and reviewed in Alexander et al., 2009). The specific presence of the homeobox gene, En1, in the 5-HT region of R1 (see below) might be another determining difference (Fox and Deneris, 2012). Finally, it was shown that Nkx2.2 deletion leads to a ventral medullar specific ablation of the GATA binding factor 3, Gata3 (Cheng et al., 2003). This specific Gata3 ablation in these 5-HT groups suggest that another regional specific Nkx2.2 dependence exists in this area.

Gata2 and Gata3 have essential function in the rostral and caudal 5-HT cell groups, respectively. In the rostral hindbrain region the combinatorial action of the Shh responsive genes Nkx2.2 and Nkx6.1 induce Gata2 and Gata3. In rhombomere1 (the rostral 5-HT neuronal group) Gata2 is sufficient to induce Lmx1b and Pet1 in the programming toward the full 5-HT phenotype. Interestingly, Gata3 is not required at this position and is unable to rescue the ablation of Gata2 (Craven et al., 2004). On the other hand, Gata3 is required for the proper specification of caudal 5-HT neurons (Van Doorninck et al., 1999; Pattyn et al., 2004). In chimeric mice of the Gata3-/- phenotype the rostral cell population seems unaltered, whereas the caudal group (mostly the nucleus raphe obscurus (ROb) group) is severely affected in terms of cytoarchitecture. Taken together, these two distinct groups of 5-HT neurons rely on two different Gata factors which suggests that these neurons rely on analogous but dissimilar gene activation programs to create the same transmitter phenotype (**Figure 1B**).

Through an elegant series of experiments involving fluorescence activated cell sorting (FACS) of 5-HT neurons and subsequent transcriptome analysis, the rostral and caudal subgroup were identified and marked by the expression of Hmx homeodomains and Hox genes respectively. The rostral groups is further divided in two by the specific expression of Engrailed (En1/2) genes in one rostral subset (R1) (Wylie et al., 2010; Fox and Deneris, 2012). With the use of a Pet1-Cre driver, postmitotic 5-HT neurons lacking En1, En2, or En1/2 were generated which showed that En1 is functionally dominant and functions in phenotypic maintenance, survival, and cytoarchitecture (Fox and Deneris, 2012). Genetic lineage analysis indicated that the rostral En1 positive group may comprise only the 5-HT cells derived from rhombomere 1 (Jensen et al., 2008). These neurons move toward more caudal positions (along the B4-9 regions in a dorsal to ventral gradient of neuronal density) and therefore 5-HT neurons along the rostral caudal axes can form homogenous genetic groups next to each other linked to their respective origin. In the same study****it was shown that R2 derived 5-HT neurons, driven through the combinatorial activation of Pet1-Flp, Rse2-Cre, and the Rosa26-dual recombinase reporter (Jensen et al., 2008), comprise the B5/8/9 regions. Using Egr2-Cre as subsets driver in similar combinations they mapped the R3/5 population toward the B5/8/9 group. Interestingly, through usage of this lineage tracing approach coupled to Nkx2.2 mutants it was shown that in Nkx2.2 mutants some cells of the B1/3 group were spared in addition to R1 derived 5-HT neurons as described earlier. This excellent lineage tracing approach has led to the definition of a spatial map 5-HT neuronal origin toward the position in the adult stage and has defined some of the molecular programming of the subsets. It might be worth while to use this setup in a combination with cell sorting and RNA-sequencing to define the exact molecular makeup of these subsets.

### CROSS DOMAIN SUBSET SPECIFICATION AND SUBSET MARKERS

The above describe subsets specification could be coupled to an anatomical location as the rostral and caudal 5-HT neurons with accompanying molecular programming. In reality there are more levels of subsets specification that do not follow these domains (**Figure 1C**), although this might in part be due to the fact that most rostral 5-HT neurons migrate to more caudal positions causing a positional mix of 5-HT subsets in the adult stage (Jensen et al., 2008). First, the 5-HT phenotype defining factor Pet1 is not essential for full differentiation of all 5-HT neurons of the raphe nucleus (Hendricks et al., 2003). In these classic Pet1-KO animals a small group of 5-HT neurons (~30%; 10–15% transmitter level) survive this ablation, almost equally distributed over the B1–B9 5-HT groups. Importantly, of these remaining 5-HT positive neurons, Aadc and 5-HT are present whereas the levels of Tph, Vmat, and Sert are diminished. In a recent study the functionality of these spared 5-HT neurons was confirmed as well as the presence of Tph2 in these neurons (Kiyasova et al., 2011). The data suggest that within the current distinction of 5-HT neuronal programming, specific differences must exist that define the specific requirement toward Pet1. An indication for differences in Pet1 dependence was found by genetic mapping of the Pet1 promoter region (Scott et al., 2005). In these experiments it was found that Pet1 acts as a maintenance factor for its own expression in a subset of 5-HT neurons, again evenly distributed for all B groups. So the activation and maintenance of Pet1 and its downstream targets, as described above, show specific dependence on Pet1.

A second not-region-defined subset refinement could be described by the selective absence of the 5-HT1A autoreceptor (Kiyasova et al., 2013). In these experiments using two reporter lines, ePet1-Gfp and 5-HT1A-iCre/R26R, they established through immunohistochemical techniques and single cell PCR that about 16% of cells measured over all 5-HT neuronal domains did not express this 5-HT1A autoreceptor. Taken together, these data suggest that another layer of molecular programming exists that defines Pet1 dependency and 5-HT1A autoreceptor presence. Although the regulation of the 5-HT1A autoreceptor has been described with some detail (reviewed in Albert, 2012), including activation by Pet-1 (Jacobsen et al., 2011), it is not clear how these processes act in concert to drive the 5-HT1A molecular distinctions. Finally, it has been described that the vesicular glutamate transporter 3 gene, Vglut3, is expressed in most but not all 5-HT neurons, located in the medial region of the dorsal (B4, B6-7) and medial (B5/B8) raphe nuclei (Herzog et al., 2004). This subset specific localization of Vglut3 was confirmed in a later, more in-depth study, which showed that co-localization (fluorescent double in situ hybridization) with Tph2 was about 80% for the dorsal raphe nucleus, with the exception of the lateral wings were the co-localization dropped to about 6% (Hioki et al., 2010). This expression pattern seems mostly limited to the rostral 5-HT cluster, suggesting that within this rostral domain additional distinctions should be present in terms of molecular programming. It might be interesting to analyze the possible link toward the presence of En1 in specific rostral subsets (**Figure 1B** and see above) and the presence of Vglut3.

Analogous immunohistochemical mapping of neuropeptides within 5-HT neurons suggested that in mice a very small number of 5-HT neurons express substance P or galanin (Fu et al., 2010), although this analysis should be completed by other approaches showing that the transcripts are present in 5-HT neurons. Taken together, these data suggest that besides the programming distinction between rostral and caudal clusters, more refinement over and within these two clusters may be present, either depending on Pet1 or on other 5-HT subset regulators.

## INNERVATION REPERTOIRE OF 5-HT NEURONAL SUBSETS

It is well known that 5-HT neuronal projections are present in almost all areas of the brain without a real specific profile. The above mentioned molecular subset specification of 5-HT neurons would suggest that specific innervation programs are present and are guided by unique programs of guidance molecule expression. Analysis of different guidance-factor mutants have shown the involvement of the frizzled (Fzd3), vang-like (Vangl2), cadherin, EGF LAG seven-pass G-type receptor (Celsr3) and Slit homolog (Slit1/2) family members (reviewed in Kiyasova and Gaspar, 2011). In Fzd3 mutants both the rostral and caudal 5-HT neurons lose their normal polarity (rostral group in rostral direction, caudal group in caudal direction) in axon outgrowth. The resulting axons are growing in all directions except for the midline. Interestingly, in Celsr3 mutants only the rostral groups seems affected in a similar manner as in the Fzd3 mutant. The Vangl2 mutant mimics the Fzd3 mutant with exception of the lateral outgrowth of the rostral 5-HT axons (Fenstermaker et al., 2010). Another study described the effects of Slit mutants (Slit2 and Slit1/2 double mutants) on the trajectory of 5-HT neurons in the brain (Bagri et al., 2002). Here, it was shown that both dopaminergic and 5-HT fibers were displaced ventrally in respect to the normal main forebrain bundle path in the Slit2 mutant. This defect is more severe in the Slit1/2 double mutant where fibers aberrantly cross the midline in ventral regions of the diencephalon and in addition the main forebrain bundle splits in two parts where some fibers run into the ventral hypothalamic area. Interestingly, in transcriptome and expression verified data of 5-HT neurons (Supplemental Table 1 in Wylie et al., 2010), Slit1 gene expression was identified, indicating that at least Slit1 is a functional component of the 5-HT neuronal axon guidance instructions set. The other above mentioned axon guidance components were not found in these data and if these data are correct it suggests that these components may not themselves be involved in 5-HT neurons, but maybe their signaling counterparts. Recently, a paper was published that described in more detail, through a lineage tracing setup (Jensen et al., 2008) the connectivity of R1, R2, and R3/5 originating 5-HT neuronal clusters (Bang et al., 2012). Although some areas like the hypothalamic region are evenly innervated by all these subsets, some areas show significant specificity in innervation pattern. The amygdala and subregion, basolateral amygdala, receive selective R1 derived innervation and R1/2 derived innervation respectively. Furthermore, the parietal cortex only receives 5-HT input from the R2 derived cluster. Also, lateral striatal areas receive selective input from the R1 cluster only. These examples show that selective axon guidance, synapse formation and pruning mechanism exist in these 5-HT subsets. What the molecular determinants are beneath this selectivity remains to be elucidated. The combination of lineage tracing techniques with FACS approaches and RNA-sequencing will in the near future solve this question.

## CONCLUDING REMARKS

The development of the 5-HT system depends on a variety of molecular programs that determine the synthesis, re-uptake and signaling of 5-HT in the central nervous system. Recent developments in this field have elucidated the core genetic program in the development of the 5-HT neurons and latest data suggest that a subdivision can be made resulting in molecular distinction between 5-HT nuclei over the rostral to caudal axis (**Figure 1B**). In the rostral cluster it is clear that the programming of the R1 sets can be distinguished from R2/3 at different levels: (1) direct 5-HT neuronal developmental programming versus fate switch from initial VMN neurons toward 5-HT neurons; (2) Influence of the Hox cluster (R2/3) compared to Hmx genes (R1) and (3) selective influence of En1 in R1 versus R2/3. The gap between 5-HT clusters in R4 is maintained through HoxB1 expression, that is important in repressing Foxa2 and thereby maintaining the VMN lineage. Finally the caudal R5-8 clusters is defined by a specific dependence on Gata3 activity suggested to be activated by Nkx2.2 in this region.

The above described subset specificity is not well understood in terms of specific connectivity. Although some studies do report initial guidance codes and specific connectivity patterns for 5-HT neuronal subsets, the exact molecular guidance code in the described subsets remain to be elucidated. The most promising data, in our view, are presented by the recent transcriptome data (Wylie et al., 2010) together with the described advanced cell-lineage analysis tools (Jensen et al., 2008). Meta analysis of these data sets might shed light on the presence of subset specific guidance codes in relation to subsets specific connectivity.

## Conflict of Interest Statement

The authors declare that the research was conducted in the absence of any commercial or financial relationships that could be construed as a potential conflict of interest.
